# Efficient Graph-Based Resource Allocation Scheme Using Maximal Independent Set for Randomly- Deployed Small Star Networks

**DOI:** 10.3390/s17112553

**Published:** 2017-11-06

**Authors:** Jian Zhou, Lusheng Wang, Weidong Wang, Qingfeng Zhou

**Affiliations:** 1 School of Computer and Information, Hefei University of Technology, Hefei 230009, China; zhoujian2009yes@126.com (J.Z.); enqfzhou@ieee.org (Q.Z.); 2 Key Laboratory of Universal Wireless Communications (Beijing University of Posts and Telecommunications), Ministry of Education, Beijing 100876, China; wangweidong@bupt.edu.cn

**Keywords:** sensor network, Internet of Things, small cell networks, resource allocation, maximal independent set

## Abstract

In future scenarios of heterogeneous and dense networks, randomly-deployed small star networks (SSNs) become a key paradigm, whose system performance is restricted to inter-SSN interference and requires an efficient resource allocation scheme for interference coordination. Traditional resource allocation schemes do not specifically focus on this paradigm and are usually too time consuming in dense networks. In this article, a very efficient graph-based scheme is proposed, which applies the maximal independent set (MIS) concept in graph theory to help divide SSNs into almost interference-free groups. We first construct an interference graph for the system based on a derived distance threshold indicating for any pair of SSNs whether there is intolerable inter-SSN interference or not. Then, SSNs are divided into MISs, and the same resource can be repetitively used by all the SSNs in each MIS. Empirical parameters and equations are set in the scheme to guarantee high performance. Finally, extensive scenarios both dense and nondense are randomly generated and simulated to demonstrate the performance of our scheme, indicating that it outperforms the classical max K-cut-based scheme in terms of system capacity, utility and especially time cost. Its achieved system capacity, utility and fairness can be close to the near-optimal strategy obtained by a time-consuming simulated annealing search.

## 1. Introduction

**Figure 1:**
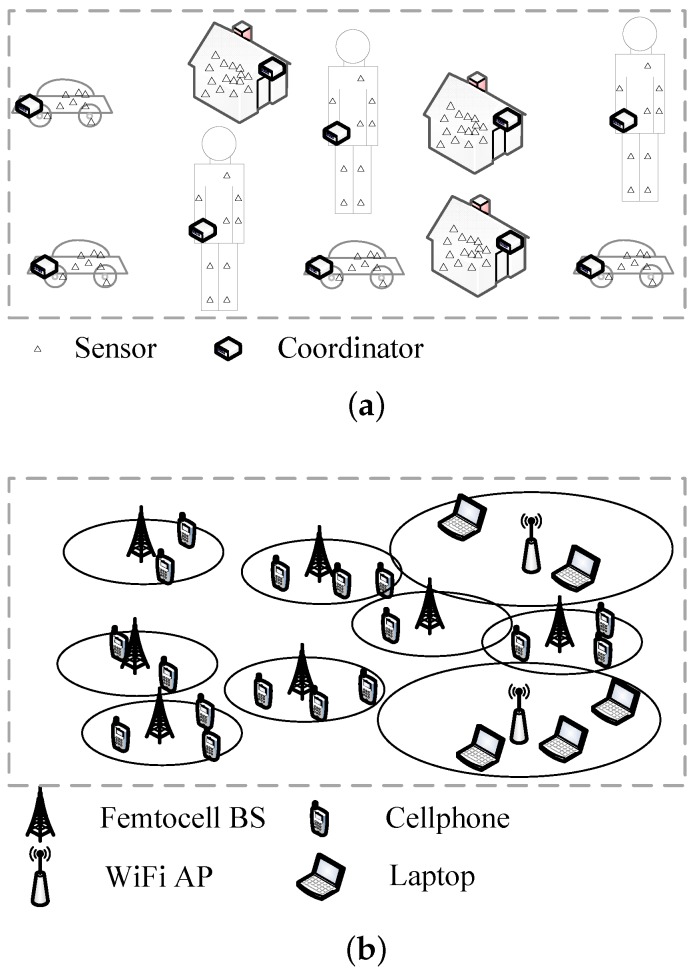
Scenarios for randomly-deployed small star networks (SSNs): (**a**) sensor clusters; (**b**) femtocells and WiFi hotspots.

Heterogeneity and high density have been important trends for the research and development of wireless communication and networking in recent years toward 5G mobile communication systems supporting future promising applications, such as artificial intelligence, smart cities and telemedicine. This leads to the mission of deploying more small access points (APs), such as WiFi hotspots and femtocells, as well as clustering of sensors into small groups for the Internet of Things (IoT) [1,2]. Therefore, many studies involve scenarios with randomly-deployed small networks, each obeying a star topology. Three typical cases are identified, i.e., femtocell, WiFi hotspot and sensor cluster, as shown in Figure 1. The first sub-figure shows the clustering of wireless sensor networks, where clusterheads are voted on based on a certain clustering scheme and sensors are divided into clusters based on a certain rule [3,4]. This example can be also extended to body sensor network (BSN) scenarios, where a coordinator organizes a BSN in the center and a group of sensors on, in or around the body are connected to it [5,6]. The second sub-figure shows a number of femtocells covering many mobile user equipment (UEs) [7,8] and a number of WiFi hotspots covering many wireless terminals [9]. Both femtocell base stations (BSs) and WiFi APs are usually deployed to form indoor local area networks for high-speed wireless access. Meanwhile, clusterheads, femtocell BSs and WiFi APs all obey random deployment, which may be modeled as a Poisson point process (PPP) [10], and they all form small star networks (SSNs) with these equipment in the center. For overall management of radio resource among these networks, centralized resource allocation schemes might be used, and entities performing such schemes could be a macro BS in cellular networks, an access controller in WLAN and a monitoring and control center for telemedicine or transportation applications in IoT. The resource, once allocated, is mainly used for transmissions inside these networks, but interference between adjacent SSNs is unavoidable and becomes the main limitation for the performance of the whole system.

Traditional resource allocation schemes are not specifically designed for randomly-deployed SSNs, so they may not perform well in these scenarios. Resource allocation for homogeneous cellular networks has been widely studied [11,12], but the deployment of macrocells is almost regular, making their schemes also quite regular. For example, various fractional frequency reuse (FFR) schemes [13], also known as static resource allocation and interference coordination, allocate frequency bands based on the division of each macrocell into multiple circles to avoid severe inter-cell interference on cell edges, so this is obviously unsuitable for randomly-deployed SSNs due to the fact that these networks are too small to be further divided into multiple circles. There are also many dynamic or semi-static schemes for cellular networks in the literature [14], as well as optimization with some well-known ideas, such as cell range expansion (CRE) and almost blank subframe (ABS) [15,16], but they need much signaling and perform periodic resource allocation, making them too time consuming for future dense networks.

Frequency point allocation for WiFi hotspots does not seem to be a new issue, but existing studies mainly focus on the allocation of a single frequency point to each hotspot and mainly consider carrier sense multiple access with collision avoidance (CSMA/CA) within each hotspot instead of scheduling-based resource allocation [17]. Both of the features above make the interference relationship between wireless terminals of adjacent WiFi hotspots different from this study. Some traditional studies on the optimization of transmissions of randomly-deployed ad hoc nodes look also similar [18,19], but transmissions are between nodes, different from the new scenarios where transmissions are inside the SSNs (the smallest unit considered in our model). To sum up, those traditional studies basically focus on resource allocation to links or contention between adjacent nodes, which does not solve the problem in SSN scenarios.

There exists some recent studies on the allocation of frequency bands to femtocells. One method is to apply representative intelligent optimization algorithms, such as evolutionary algorithms, to search for an allocation solution with high performance [20,21], but these algorithms always need a very long time to find a near-optimal solution, which is unsuitable for usage in future networks with dense traffic and massive devices. Graph theory is a common tool for similar topics because it fits random deployment scenarios, and graph-based algorithms usually achieve much lower time complexity than intelligent optimization algorithms. Studies using graph theory are summarized as follows. Some studies built interference graphs and modeled the issue as max K-cut (MKC) problems. Sadr et al. considered resource allocation in femtocell networks as a graph coloring problem using the interference graph and proposed a scheme based on MKC [22]. Chang et al. also modeled downlink resource allocation as an MKC problem using the interference graph and proposed a two-phase scheme containing a greedy-like MKC searching in the first phase and a heuristic algorithm in the second phase [23]. The proposed scheme had relatively low complexity, but still too high for dense and massive networks. Moreover, the scenario studied in [23] was homogeneous networks, and the interference graph was established for users, while the scenarios considered in this article require building the interference graph for SSNs and consider transmissions inside SSNs. Pateromichelakis et al. studied resource allocation for small cell networks using the interference graph, obtained the optimal solution using the branch-and-cut approach and proposed a heuristic scheme based on a minimum path selection procedure [24].

Some papers, instead of using the interference graph, built the contention graph to solve the contention among links formed by randomly-deployed femtocells, and the maximum weighted independent set (MWIS) was used as a tool for a group of femtocells to separate the allocation of resources [25]. Chen et al. applied the contention graph and maximum independent set to study the opportunistic link scheduling, which achieved a distributed graph theory-based algorithm with the optimal policy for IEEE 802.11 CSMA/CA networks. The contention graph is suitable for solving the contention among links formed by randomly-deployed femtocells [26], but it may not be suitable for the issue studied in this article. That is because, each vertex in a contention graph represents a link, and an MWIS indicates a group of links that can simultaneously transmit, which is different from transmissions inside the SSNs in this issue. Moreover, the searching of MWIS is NP-hard [18], while the issue in this article demands a very low-complexity scheme.

The above discussion demonstrates that, along with the development of wireless communication systems toward heterogeneous networks with dense traffic and massive devices, instead of looking for the optimum or near-optimum, the main demand of resource allocation in these scenarios is a very low-complexity scheme with relatively good performance. Meanwhile, the consideration of interference for SSNs is different from existing studies for nodes. The allocated resource is mainly used for transmissions inside each SSN, so the transmission (also interference) range is relatively small. For example, sensors in a BSN transmit with low power, so they only interfere with a small number of BSNs quite close to them. By contrast, the interference range of a node in previous studies is usually large, depending on the distance to its destination, so it interferes with a large number of adjacent nodes. This difference affects the constructed interference graph, obviously leading to less edges. Therefore, resource reuse for SSNs is more prominent, which further stimulates our following consideration of the MIS-based design. In detail, existing MWIS-based ideas lead to too high of a time complexity in these scenarios, while MKC-based ideas are relatively faster. For example, in [23], a complete MKC-based scheme is proposed and evaluated, which is a quite representative work among similar studies and thoroughly compared with our proposed scheme later. To further improve the system performance, especially the time complexity, in this article, the interference graph is used to model the scenarios in Figure 1, and the unweighted maximal independent set (MIS) is applied, whose time complexity for one search is only O(log(N)); see the detailed description in Section 3. Then, an MIS-based resource allocation scheme is proposed, which divides the vertices of the graph into multiple kinds of sets (such as leader set, leader adjacent set and MISs) and designs empirical resource allocation strategies for them. Extensive simulations show that the proposed scheme is very efficient compared with the MKC-based scheme. Its performances, in terms of system capacity and utility, are also significantly improved. Its achieved fairness, in terms of Jain’s fairness index, is not much worse, especially when a large α parameter is set in empirical equations of the scheme. Meanwhile, the performance in terms of the above metrics is close to the near-optimal strategy obtained by a time-consuming simulated annealing search, further indicating that it is worth suffering a small possible decrement of fairness as long as a prominent increase of efficiency is achieved without obviously degrading system capacity and utility.

The remainder of this article is organized as follows. Section 2 provides the system model. Section 3 contains three subsections explaining interference graph construction, MIS searching and the proposed scheme. In Section 4, extensive simulations are performed to demonstrate the performance of our proposal in both dense and nondense scenarios. Finally, the article is concluded in Section 5.

## 2. System Model

Let us consider the scenarios in Figure 1. To unify the description in the following sections, sensor clusters, femtocells and WiFi hotspots are called SSNs uniformly (as defined in the previous section). These networks are the smallest units for resource allocation, while the consideration of further allocation inside these networks is not within the scope of this study. Therefore, a unified scenario containing *N* randomly-distributed SSNs in a square area is used for modeling the system. We only consider transmissions inside each SSN, while interference happens in between.

For the resource allocation issue, the resource could be time slots in the time domain and frequency points (or groups of subcarriers) in the frequency domain, accordantly called resource blocks (RBs). Given *M* RBs in a wireless communication system, each SSN is assigned a subset, written as:

(1) $$assignment_{i}=\left\{a_{ij}|j=1,2,\ldots ,M\right\},$$

where *i* indicates the index of the SSN, *j* indicates the index of the RB and $a_{ij}$ is a binary variable indicating the usage status of RB *j* by SSN *i*, given by:

(2) $$a_{ij}=\left\{\begin{array}{cc} 1 & \mathrm{use}\;\mathrm{the}\;\mathrm{RB},\\ 0 & \mathrm{otherwise}. \end{array}\right.$$

Let us consider the total interference on SSN *i*. Any other SSN using the same RBs might be an interferer, so the total interference received by SSN *i* is written as:

(3) $$I_{i}=\sum_{l=1,l\neq i}^{N}\left(\frac{1}{M}\sum_{j=1}^{M}a_{ij}\cdot a_{lj}\right)\cdot I_{il},$$

where $I_{il}$ is the point-to-point interference value between SSN *i* and SSN *l*, related to the distance in between. Note that interference from equipment outside of this system (such as from a macrocell to femtocells or from Bluetooth equipments to WiFi) is not integrated into this model. There are two reasons that force us to make this assumption: on the one hand, those equipment’s deployment can be considered independent of this system, so the interference effect tends to be equivalent to different SSNs; on the other hand, interference coordination among heterogeneous networks has been widely studied [15,16,27] and the modeling of such a kind of interference should be highly correlated with those coordination schemes, making it quite difficult to evaluate the real interference and to combine it in this model. Thus, the signal-to-interference-plus-noise ratio (SINR) of SSN *i* can be written as:

(4) $$SINR_{i}=\frac{P_{t}}{I_{i}+N_{0}},$$

where $P_{t}$ is the transmitting power and $N_{0}$ is the variance of additive white Gaussian noise (AWGN). Hence, the capacity of SSN *i* is written as:

(5) $$C_{i}=\sum_{j=1}^{M}a_{ij}\cdot Bd_{per}\cdot \log_{2}\left(1+SINR_{i}\right),$$

where $Bd_{per}$ is the minimum unit of bandwidth for allocation. Finally, the objective of this study is to optimize the total capacity of all the SSNs written as:

(6) $$C_{total}=\max\sum_{i=1}^{N}C_{i}.$$

Taking fairness into consideration, the objective of the above optimization becomes:

(7) $$U_{total}=\max\sum_{i=1}^{N}U_{i},$$

where $U_{i}$ is the total utility of SSN *i* integrating α fairness [28], given by:

(8) $$U_{i}=\left\{\begin{array}{cc} \ln C_{i} & \alpha =1,\\ \frac{C_{i}^{1-\alpha }}{1-\alpha } & \alpha \neq 1. \end{array}\right.$$

α fairness is a well-known generic way to integrate fairness consideration into an objective function. α=0 represents the previous objective function on capacity; α=1 represents proportional fairness; and α=+∞ represents max-min fairness. Note that the problem falls into the category of combinatorial optimization with variables $a_{ij}$ indicating the assignment of RBs to the SSNs. In this issue, each SSN could use multiple RBs, and each RB could be used by multiple SSNs, so this optimization issue does not have any specific constraint except that (2) indicates the binary variables $\left\{a_{ij}\;|\;1\;\leq i\;\leq \;N,1\leq j\leq M\right\}$ in the issue. Note that, instead of solving a specific optimization issue by a certain technique such as constraint relaxation, the purpose of this article is to propose a scheme that has very low complexity, so that it is fast for the scenarios with massive SSNs to reach a relatively good solution. Therefore, the optimization issue defined above is a generic issue of resource allocation for SSNs containing $2^{M\times N}$ solutions. The optimality is highly related to the interference values $I_{il},i,l\in \left[1,N\right],i\neq l$. For any SSN *i* and SSN *l*, if $I_{il}$ is small enough, the two SSNs can use the same RBs. Moreover, since each SSN could use multiple RBs, all the RBs might be allocated to some SSNs under weak interference, resulting in an unfair allocation. Therefore, the usage of α fairness in the objective function could lead to fairer allocation to the SSNs under severe interference.

## 3. The Proposed Graph-Based Scheme

### 3.1. Interference Graph

When two SSNs use exactly the same RBs for transmission, there might be interference between them. The strength of this interference highly depends on the distance between the two SSNs. When the distance is large enough, this interference might be ignored, so there might be a distance threshold $d_{th}$ to indicate whether the interference is ignorable or not. This threshold might be disparate for different scenarios or different objective functions of optimization, but this difference would not obviously affect the following study in this article. That is because, with different thresholds, the difference on the interference graph might be only a few edges added or removed. The changes usually happen at the places with dense deployment, which further causes only a very slight effect on the obtained MIS in the following subsection and the final performance, so the comparison of different ways to derive the distance thresholds and the discussion of the best distance threshold are out of the scope of this article. For the following examples and for performance evaluation, the distance threshold derived in the Appendix is used, which is obtained in a two-SSN scenario with the objective of maximizing their total capacity. Since this threshold is derived in a two-SSN scenario whose interference is less than dense scenarios, it can be considered as a relaxation that leads to an interference graph with more edges. Therefore, this relaxation only increases the time cost of the following procedures without changing its optimality feature.

**Figure 2:**
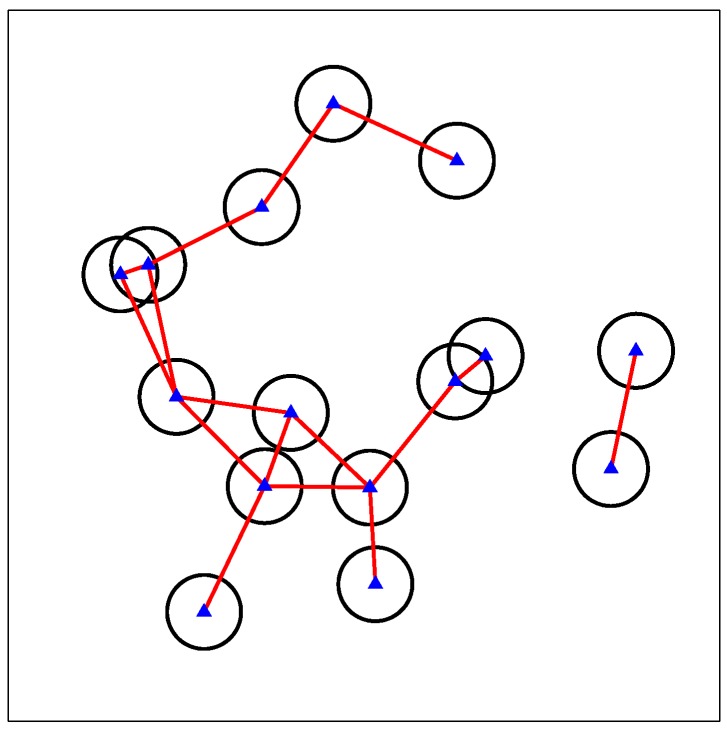
A constructed interference graph.

Once a distance threshold is given, an interference graph can be constructed for a certain scenario [29], whose vertices represent the SSNs. A matrix E can be used to describe the edges, whose elements are defined as:

(9) $$e_{il}=\left\{\begin{array}{cc} 1 & d<d_{th},\\ 0 & d\geq d_{th}, \end{array}\right.$$

where $e_{il}$ is a binary variable with i,l∈[1,N] and i≠l, indicating whether the interference between SSN *i* and SSN *l* is ignorable ($e_{il}=0$) or not. If it is ignorable, no edge between vertex *i* and vertex *l* exists in the graph; otherwise, an edge connects the two vertices. Taking Figure 2 as an example, a number of randomly-deployed SSNs form an interference graph with edges connecting the vertices suffering un-ignorable interference. The graph might be divided into multiple isolated subgraphs due to the distances in between, but there is no difference for the following process. We can process each isolated subgraph separately or together during the calculation of MISs and the resource allocation proposed in the following section, leading to the same results.

### 3.2. MIS

For two SSNs far from each other, since their inter-SSN interference is small, the total capacity can be increased by using the same RBs repetitively. The interference graph described in the above subsection is a subset of the complete graph, whose edges corresponding to such a case are removed. Therefore, in order to increase the total capacity, vertices unconnected in an interference graph can use the same RBs. It is an MIS problem to find a maximal subset of vertices that are unconnected in an interference graph [30,31]. In this subsection, we describe two methods to obtain one or multiple MISs: (1) a theoretical method obtaining all the MISs; (2) a very efficient algorithm obtaining one MIS.

Given an interference graph G, we use V(G) to represent the set of vertices, E(G) to represent the set of edges and |·| to represent the number of vertices in the graph. An independent set is a subset of V(G) subject to the restriction that neither of any two vertices in this subset are connected by an edge.

**Definition** **1.** *For an independent set* S *of a graph* G*, if it is not a subset of any other independent sets of* G*, it is called a maximal independent set (MIS).*

We say that an edge of a graph G is covered by a subset of V(G) if at least one of the two vertices of this edge belongs to the subset. A subset of V(G) is called a cover set of V(G) if all the edges in E(G) are covered.

**Definition** **2.** *For a cover set* K *of a graph* G*, if none of its subsets is a cover set of* G*, it is called a minimal cover set.*

**Figure 3:**
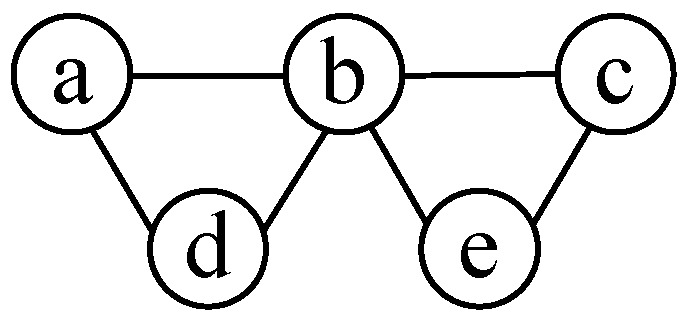
An example for maximal independent set (MIS) calculation.

As basic knowledge in graph theory, MISs and minimal cover sets have a one-to-one complementary relationship, i.e., for any minimal cover set β(G), V(G)−β(G) is an MIS [32]. Therefore, one way to obtain all the MISs for a graph is to find all the minimal cover sets and to convert to their complementary sets. Given a graph, we firstly mark for each vertex the adjacent vertices connecting with it, called a connection indicator of this vertex. Secondly, connection indicators of all the vertices are multiplied and transformed into a polynomial of multiple terms by polynomial expansion, each term representing one minimal cover set. Thirdly, we take the inversion of each term to obtain all the MISs. We can also finally obtain the maximum independent set(s) by choosing the one(s) with the maximum number of vertices. Note that MIS and the maximum independent set are two different concepts. The latter corresponds to the MIS(s) with the largest number of vertices among all the MISs. Take the graph in Figure 3 as an example, the connection indicator of vertex *a* is written as (a+bd), that of *b* is (b+acde), and so on. The multiplication of all the connection indicators is given by:

(10) (a+bd)(b+acde)(c+be)(d+ab)(e+bc)=acde+abe+bcd+abc+bde.

There are five terms in the above polynomial, corresponding to five minimal cover sets, hence five MISs. For example, acde in (10) is a minimal cover set, and its inversion is *b*, an MIS. By comparing the number of vertices in each MISs, we find that the 2nd, 3rd, 4th and 5th terms in (10) all correspond to maximum ones, i.e., cd, ae, de and ac.

The above method obtains all the MISs, but the time cost is non-polynomial. Considering that our resource allocation scheme does not need all the MISs, the following very efficient algorithm will be used to obtain one MIS at once, which is an improvement from the algorithm described in [30] to make it faster for obtaining one random MIS. The algorithm starts from the set of vertices V, and for each round, a random vertex *i* is chosen and put into the MIS. If it is not an isolated vertex, the adjacent vertices should be removed from the potential MIS, corresponding to Line 7 in the algorithm. The improvement is the removal of vertices’ profits and corresponding comparisons to choose vertices with lower profits during the form of an MIS. In detail, the original algorithm in [30] defines a profit value for each vertex, so that it will be used to choose the one with lower profit when any two adjacent vertices are compared. Therefore, that algorithm obtains an MIS with relatively low total profit for a weighted graph where vertices have profits. By contrast, the algorithm we need to use later for MIS searching in our proposed scheme is for a graph without vertex profits, so we simplify that algorithm by removing the considerations and comparisons of vertex profits to further save running time. This improvement concerns the implementation detail, which does not decrease its complexity.

**Algorithm 1** Obtaining one random MIS.**Input:**
V, E **Output:**
MIS1:**Initialization:** V→MIS, adjacent(i)={j∈V|(i,j)∈E}2:**while** V is not empty **do**3:  vertex *i* is randomly chosen from V4:  **if** adjacent(i) is empty **then**5:    remove *i* from V6:  **else**7:    remove adjacent(i) from MIS8:    remove *i* and adjacent(i) from V9:  **end if**10:  return MIS11:**end while**


### 3.3. Proposed Scheme

In order to apply the MIS concept for designing an efficient scheme, we randomly generate extensive scenarios, use the simulated annealing algorithm to study their near-optima and summarize the following highlights:

(1) Adjacent SSNs (i.e., those connected by edges in the interference graph) should generally not use the same RBs; otherwise, severe interference decreases all their capacities. For each SSN, since its capacity is decreased, its utility integrating α-fairness should be also decreased because of the monotonous feature of the α-fairness function with regard to capacity. Since an MIS provides a set of unconnected vertices in a graph, we could allocate the same RBs to an MIS and repetitively use them for all the SSNs in it. Different MISs should not use the same RBs due to the fact that they are definitely connected by some edges.

(2) When we consider a small α value, more RBs should be allocated to the SSNs under relatively slight interference, so that the total capacity of the whole system is high. Vertices on the border of an interference graph usually have only a small number of edges, so SSNs on the border of the scenario achieve high capacity on average and contribute more to the system capacity than those in the center.

Based on the above analysis, we believe that a certain ratio of SSNs on the border should be chosen and allocated more RBs than the others. However, due to the randomicity of the scenario, we cannot predict the features of interference to fix an allocation strategy. Therefore, to choose a number of vertices on the border, we set a vertex percentage parameter γ and gradually add the vertices to an alternative set starting from those with the smallest number of edges till the percentage of vertices in this set exceeds γ. Some vertices in this set might be connected by edges, indicating that they should not use the same RBs. To find a subset of vertices unconnected with one another from this set, an MIS is obtained as the chosen set of vertices on the border of the graph. Considering that SSNs corresponding to this set contribute significantly to the system capacity, we call it the leader set. Note that the search for this MIS starts from the vertices with a smaller number of edges, so that an MIS under almost the slightest interference is obtained.

For the ratio of RBs used by the leader set, we have the following observations. When α=0, fairness is not considered, so the ratio is always 100% to maximize the system total utility. Along with the increment of α-fairness, the ratio of the allocated RBs to the leader set decreases, and we find that the relationship between this ratio and α is quite close to a sigmoidal function. When we use a large α value, RBs will be allocated in a fairer way, but the ratio for the leader set has a maximum degradation around 1/4 due to the tradeoff between utility and fairness. That is because of its inherent low-interference feature, i.e., the interference for SSNs in the leader set is always less severe than the SSNs in the center of a scenario. Based on the above analysis, the following empirical equation and corresponding parameter values for the sigmoidal function are obtained to calculate the ratio of RBs for the leader set:

(11) $$\mathrm{RB\_Leader}=1-\frac{1}{4}\cdot \frac{{\left(\frac{\alpha }{0.5}\right)}^{3}}{1+{\left(\frac{\alpha }{0.5}\right)}^{3}}.$$

(3) Based on the first observation above, adjacent vertices should generally not use the same RBs. Adjacent vertices of the leader set include those in the above alternative set, but not chosen as leaders, and those connected with the leaders, but not in the alternative set. We put all these vertices into a set, called the leader adjacent set. Considering that these vertices are close to the border of a graph, they are on average under slighter interference than the vertices in the center, so they should use as much resource as possible. Since the leader set has occupied most of the RBs, we assign all the remaining RBs to the leader adjacent set without retaining any for other usage, but how to use the remaining RBs in the leader adjacent set should be considered carefully due to the fact that some vertices in the leader adjacent set might be connected (interfered) with one another.

On the one hand, since the vertices in the leader adjacent set themselves also form a complex graph, it is time consuming to find the optimal or a near-optimal allocation strategy of RBs in the leader adjacent set by certain combinatorial optimization approach or other optimization theories. Note that for all the other sets (leader set and the MISs in the central part of the graph), the optimization approach was not used either. We design their allocation strategies based on our experience with extensive empirical results, which is the main reason and merit to achieve a fast scheme. Therefore, we should not use certain optimization approaches to orthogonally assign the RBs to some connected (interfered) vertices in the leader adjacent set; otherwise, the time cost of this part may severely slow down the speed of the whole scheme.

On the other hand, even if a long-time search algorithm is used, the performance does not improve much compared with allocating all the remaining RBs to all the vertices. This phenomenon is more obvious when the number of vertices is moderate, i.e., when the vertices in this set are not severely interfering with one another. As we know, SSNs are networks, not devices, so the density of SSNs could be moderate even for a hyper-dense network. Each SSN may be connected with adjacent ones, but the coverage of an SSN does not usually span several layers of other SSNs. Therefore, for a hyper-dense network, the density of the interference graph formed by SSNs is moderate, not hyper-dense, while for a moderate graph, connected vertices in the leader adjacent set are not many. Meanwhile, for the case when two or more vertices are connected with one another, system fairness may be obviously decreased if we give up some of them, while the system capacity may only increase a little.

For all the reasons above, we find that a simple idea allocating all the remaining RBs to all the vertices in the leader adjacent set usually corresponds to high performance, so we do not use any specific algorithm to further separate the vertices in the leader adjacent set into different sets or design different strategies for different vertices in this set.

(4) Vertices in the center of a graph usually connect to multiple adjacent vertices, so they are generally all severely interfered. Meanwhile, they interconnect and are stochastically consistent with one another, making it not so meaningful to design a detailed, but time-consuming resource allocation strategy. Therefore, we design an efficient strategy based on MIS for the vertices besides the leader set and the leader adjacent set. Note that, in dense scenarios, the number of SSNs may be very large, leading to the division of a large number of groups for the central SSNs. To guarantee a low time cost, we set a predefined parameter to indicate the maximum number of MISs Max_MISs for central SSNs, so the unallocated SSNs exceeding this value will be put together as a final group and use all the remaining RBs repetitively. Since these groups contain different numbers of vertices, we design different strategies to allocate RBs to them. For the ratio of RBs used by the first group, the observations are basically the same as for the leader set, except that the parameters in the sigmoidal function are not the same. Meanwhile, we find that the ratio of RBs for the first group is also affected by the total number of groups, and the relationship can be represented by changing the degradation from 1/4 in (11) to 1−1/q, where *q* is the number of groups we finally obtain for central vertices during the running of the scheme. The following empirical equation and corresponding parameter values for the sigmoidal function are obtained to calculate the ratio of RBs for the first group:

(12) $${\mathrm{RB\_Group}}_{1}=1-\left(1-\frac{1}{q}\right)\frac{{\left(\frac{\alpha }{0.35}\right)}^{3.5}}{1+{\left(\frac{\alpha }{0.35}\right)}^{3.5}},$$

indicating that the allocated RBs to the first group should decrease if there are more groups awaiting for resource. The α-fairness index is used to adjust the allocation so that less RBs are allocated to the first group if α is increasing. Denote the number of vertices in the *m*-th group as $n_{m},m\in \left[2,q\right]$; the ratio of RBs used by the *m*-th group is given by:

(13) $${\mathrm{RB\_Group}}_{m}=\frac{n_{m}}{\sum_{m=2}^{q}n_{m}}\left(\mathrm{RB\_Leader}-{\mathrm{RB\_Group}}_{1}\right).$$

We can see that the total RBs used by the vertices in the center of the graph are those used by the leader set. The first group for the central vertices uses more RBs than the others based on (12), while the others proportionally use the remaining RBs according to their number of vertices based on (13). One reason to allocate more RBs to the first group is that its number of vertices is obviously larger than the others. Another important reason is that, for small α, almost all the RBs tend to be allocated to the largest group, which could significantly improve the system capacity and utility.

**Figure 4:**
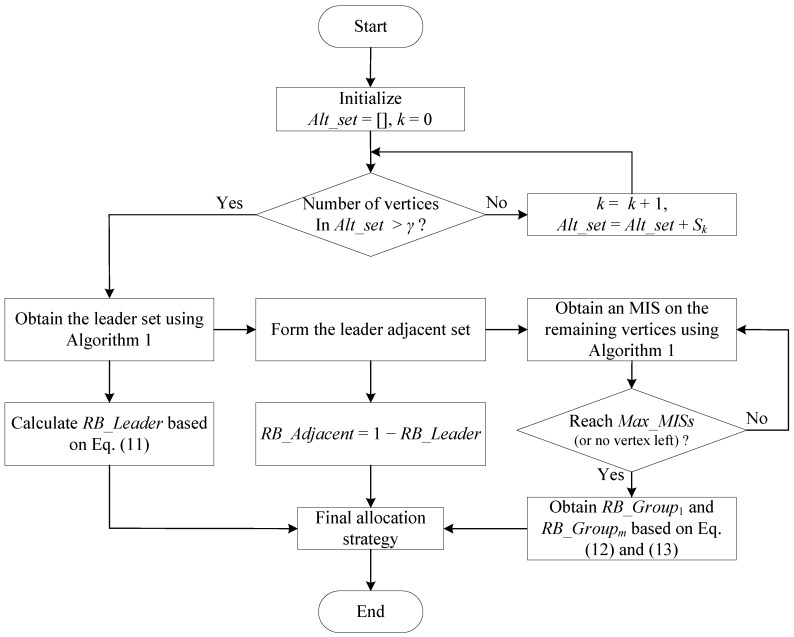
Flowchart of the proposed scheme.

Combining all the above analysis and design, we propose an MIS-based resource allocation scheme for randomly-deployed dense SSNs as follows. Inputs of the scheme include the interference graph, the vertex percentage parameter γ for the leader set and the maximum number of MISs for central SSNs Max_MISs. Based on the interference graph, we count for each vertex the number of edges and obtain a series of sets of vertices, given by $S_{k},k=1,2,3,\ldots ,N$, where $S_{k}$ contains the vertices having *k* edges. The procedure of the scheme is shown in Figure 4.

Step 1: We initialize the alternative set Alt_set as empty and the index k=0, which will be used to help obtain the final Alt_set.

Step 2: We form the alternative set by gradually adding vertices from those with the smallest number of edges, i.e., $\mathrm{Alt\_set}=\mathrm{Alt\_set}+S_{k}$ with *k* gradually increasing. A final alternative set is formed when the number of vertices in it is larger than the vertex percentage parameter γ.

Step 3: Algorithm 1 is performed on Alt_set to obtain an MIS as the leader set, and RB_Leader is calculated based on (11).

Step 4: The leader adjacent set is formed by adding all the vertices adjacent to the vertices in the leader set, and its RB ratio is obtained as RB_Adjacent=1−RB_Leader.

Step 5: We run Algorithm 1 on the remaining vertices (that are not in the leader set or the leader adjacent set), so that they are divided into multiple groups. This division loop stops when the obtained number of MISs reaches Max_MISs (or when there is no vertex left, although this does not usually happen for dense scenarios).

Step 6: Based on the division in Step 5, (12) and (13), we obtain ${\mathrm{RB\_Group}}_{1}$ and ${\mathrm{RB\_Group}}_{m},m\in \;\left[2,q\right]$; hence, the final allocation strategy is obtained for all the sets.

## 4. Performance Evaluation

In this section, we simulate the proposed MIS-based scheme and compare it with several representative schemes. Based on the analysis in Section 1, the classical MKC-based scheme in [23] is considered as a milestone scheme for comparison due to its good system performance with low complexity, which is denoted as the “Classical MKC-based scheme” in the following figures. Meanwhile, it is very interesting to know the near-optimal solutions and to check the gap between our solution and the near-optimum. We use an optimum search algorithm for combinatorial optimization issues, i.e., the well-known simulated annealing algorithm, to reach a near-optimal solution by a very long-time search, denoted as “SA for near-optimal utility” in the figures. Random allocation, just as its literal meaning, is to randomly allocate resources without any specific concern, so it does not cost any time to do any computation for resource allocation. It is interesting to see whether the performance improvement of the proposed scheme from a random allocation is worth its time cost. We consider two types of scenarios: one is nondense with a limited number of SSNs to compare the performance of all the above schemes and also to show the effect of integrating α-fairness into our proposal, while the other is dense with a large number of SSNs to compare the time cost between our proposal and the classical MKC-based scheme. One reason for considering two types of scenarios is to show the effectiveness of our scheme in both types of scenarios. Another reason is that the simulated annealing algorithm to search for a near-optimal strategy is intolerably time consuming to be used for comparison in dense scenarios. For both types of scenarios, the evaluated performance metrics include system capacity, utility and Jain’s fairness index. Moreover, we show 95% confidence intervals for most of the points in the following figures, excluding those whose confidence intervals are too small to be visible.

For the following simulations, the transmitting power of the SSNs is 21 dBm [33]; AWGN is $10^{-10}$ watts; and the bandwidth for each SSN is set to a unit value of 1 MHz. Since SSNs are usually local and indoor networks, we choose the close-in free space reference distance path loss (CI-FSPL) model for propagation, given by [34]:

(14) $$PL\left(f,d\right)=FSPL\left(f,1m\right)+10b\log_{10}\left(\frac{d}{1m}\right)+X_{\sigma }^{CI},$$

where *b* is the path loss exponent set to 3.19, $X_{\sigma }^{CI}$ represents the shadowing effect set to 8.29 dB and *f* is the carrier frequency set to 2.4 GHz in the simulations. 1m in (14) represents the unit distance for the calculation of propagation, given by:

(15) $$FSPL\left(f,1m\right)=20\log_{10}\left(\frac{4\pi f}{c}\right),$$

where *c* is the speed of light. By setting the receiver sensitivity to $10^{-11}$ watts, also combining with the above propagation model and the transmitting power, the coverage radius of each SSN can be obtained as 44.76 m.

### 4.1. Results in Nondense Scenarios

**Figure 5:**
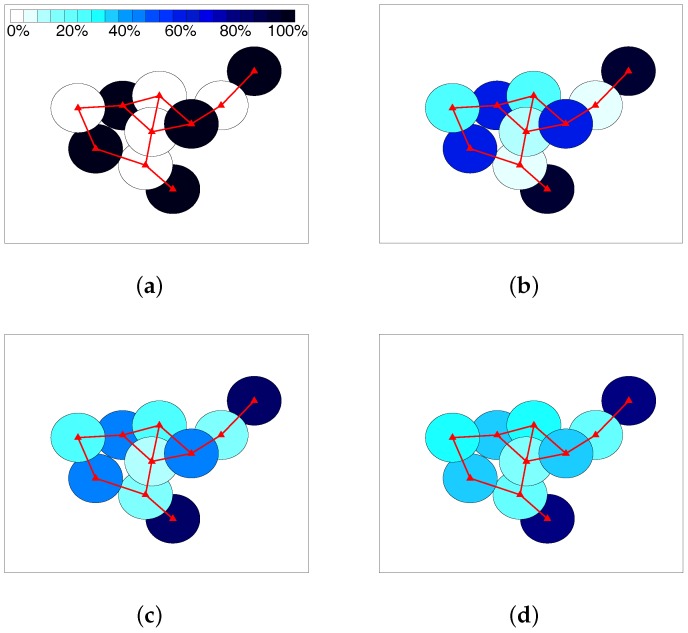
Allocation example using a randomly-generated scenario: (**a**) α=0; (**b**) α=0.4; (**c**) α=0.8; (**d**) α=1.

When α-fairness is not considered, i.e., α=0, the objective function in the system model becomes the maximization of system capacity. As is common knowledge, it will make the allocation unfair to the SSNs under strong interference. In other words, all the RBs tend to be allocated to the one under lower interference for any two SSNs connecting with each other. In Figure 5, allocation results in a randomly-generated scenario are visually shown for α equaling 0, 0.4, 0.8 and 1, respectively. The colors from light to dark represent the ratio of RBs used for a certain SSN, as shown by the color strip in Figure 5a. When α=0, all the RBs are allocated to a number of SSNs unconnected with one another, which actually form an MIS of the graph. This allocation is unfair for the SSNs not in this MIS, but maximizing the system capacity. Along with the increment of α, the ratio of used RBs by the SSNs in the MIS gradually decreases, while that of the SSNs not in the MIS gradually increases. When α reaches one, i.e., the well-known proportional fairness position, the allocation becomes relatively balanced, although the SSNs under lower interference still tend to use more RBs.

We randomly generate 100 scenarios with the numbers of SSNs between 10 and 30. For each scenario, the proposed scheme, the classical MKC-based scheme and the random allocation scheme are simulated to obtain the average capacity, utility, Jain’s fairness index of average capacity and Jain’s fairness index of utility. For each metric above, each point in a curve is obtained by averaging for the 100 scenarios, so the curves are smooth and representative. Since the numbers of SSNs are variant for different scenarios, the average capacity and utility are calculated as average values per SSN. The MKC-based scheme requires dividing the SSNs into multiple groups. For each scenario, we first run our scheme and record the number of groups that our scheme obtains. Then, the same number of groups is set to the MKC-based scheme, which ensures the rationality of the comparison. For the simulated annealing search of a near-optimal utility, due to the fact that this optimization search is quite time consuming, we randomly choose 10 scenarios from the 100 generated scenarios above, and each point in its curves is obtained by averaging these 10 scenarios. Therefore, the confidence intervals for the curves of “SA for near-optimal utility” seem larger than the other schemes, but it does not affect the comparison between different schemes, especially between the proposed scheme and the classical MKC-based scheme.

**Figure 6:**
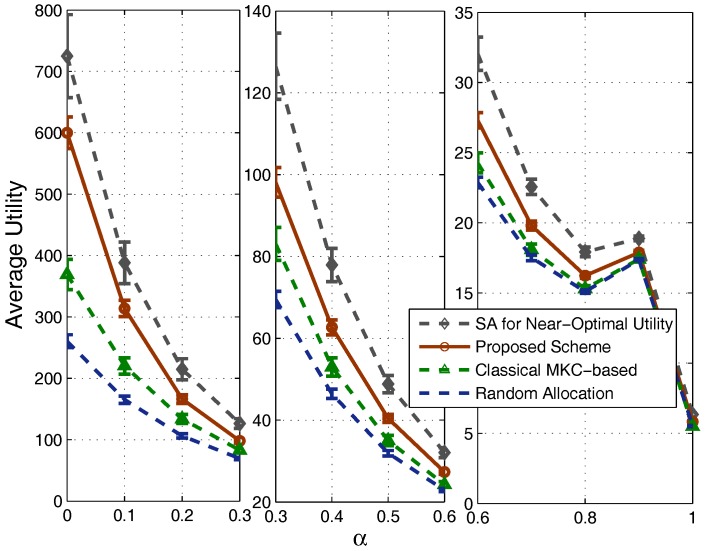
Comparison of utility.

**Figure 7:**
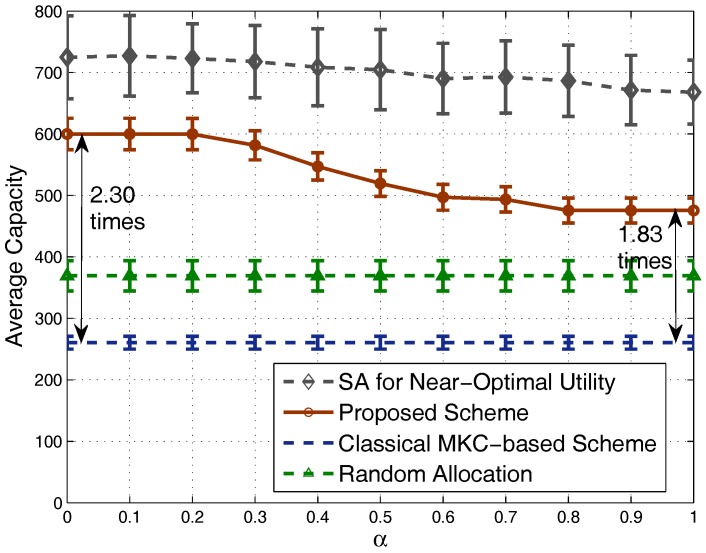
Comparison of average capacity.

Comparisons of the system capacities and utilities of the four schemes are shown in Figure 6 and Figure 7, respectively. We can see that, along with the increment of α, the utility value gradually decreases, and there is a fluctuation when α is close to one. These are actually common features of the utility function defined by (8) in the system model. An interesting observation from this figure is that the utility of our scheme is quite close to the near-optimum obtained by the simulated annealing algorithm. Note that the simulated annealing algorithm uses quite a long period to search for this near-optimal strategy, while our scheme is a quite fast heuristic one, so the advantage of our scheme is obvious. Compared with the MKC-based scheme and the random allocation strategy, our scheme achieves a much higher utility. Note that, we divide the figure into three subparts, i.e., [0,0.3], [0.3,0.6] and [0.6,1], so that the difference between the curves is still clear for the large α case.

For the capacities shown in Figure 7, we can see that our scheme achieves a quite high system capacity, close to the performance of the strategy obtained by the simulated annealing algorithm especially when α is small. Meanwhile, our scheme outperforms significantly the MKC-based scheme and the random allocation strategy. Specifically, when α is small, our scheme’s achieved system capacity is around 2.30-times that of the MKC-based scheme. When we set a large α value (e.g., α=1) to balance between system capacity and fairness, the achieved system capacity is lower than before, but still around 1.83-times that of the MKC-based scheme. Note that two of the evaluated schemes (MKC-based scheme and random allocation strategy), do not consider the α-fairness factor, and capacity is independent of α, so their achieved system capacities have a stable behavior for different α values.

**Figure 8:**
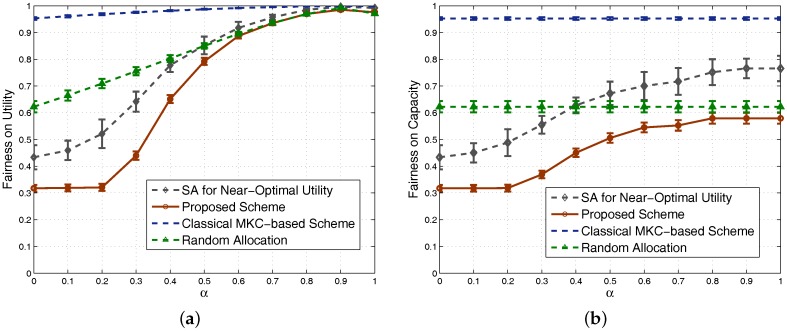
Comparison of fairness: (**a**) based on utility; (**b**) based on capacity.

Since the α-fairness factor is integrated into our utility function, fairness should be improved if a large α is used, so the well-known Jain’s fairness index is used to evaluate the achieved fairness of our proposal. Jain’s fairness index is defined as [35]:

(16) $$J=\frac{{\left(\sum_{i=1}^{N}R_{i}\right)}^{2}}{N\cdot \sum_{i=1}^{N}R_{i}^{2}},$$

where $R_{i}$ is the performance of the *i*th SSN, such as its capacity or utility. Based on this equation, we see that fairness is proportional to *J*, a value between zero and one. We calculate Jain’s fairness values for both utility and capacity, shown in Figure 8a,b, respectively. The classical MKC-based scheme achieves very high fairness, which is an important reason for us to integrate the α-fairness factor into our proposed scheme. We can see that, by using a large α, the achieved Jain’s fairness of our scheme can be significantly improved. Especially for the fairness of utility in Figure 8a, it is finally quite close to the MKC-based scheme for a large α. Combined the truth that our scheme achieves higher system capacity and utility than the MKC-based scheme shown in Figure 6 and Figure 7, our scheme has obvious benefits. Note that the main purpose of this study is to design a very fast scheme achieving relatively high performance, and we will show that our scheme significantly saves time compared with the MKC-based scheme in the simulations of Section 4.2 later. Moreover, considering the sigmoidal functions used in the proposed scheme for calculating the ratios of RBs used, the obtained ratios are close to one another when α is too small or too large, so the curves of our scheme in Figure 7 and Figure 8 all look like a sigmoidal form.

### 4.2. Results in Dense Scenarios

This subsection focuses on the dense scenarios where 30–100 SSNs are randomly deployed in a small simulation area. Since the coverage radius is set to 44.76 m based on the settings described at the beginning of the section, we set the simulation area to 500 m × 500 m, so that the SSNs could severely overlap with one another. To obtain relatively smooth curves, we generate 50 random scenarios for each given number of SSNs, and each point on a curve below is obtained by averaging the performance of the 50 scenarios. Since the time cost of the simulated annealing algorithm dramatically increases when the number of SSNs is large, the purpose of this subsection is to compare our proposal with the MKC-based scheme to demonstrate the benefits of our proposal in dense scenarios.

**Figure 9:**
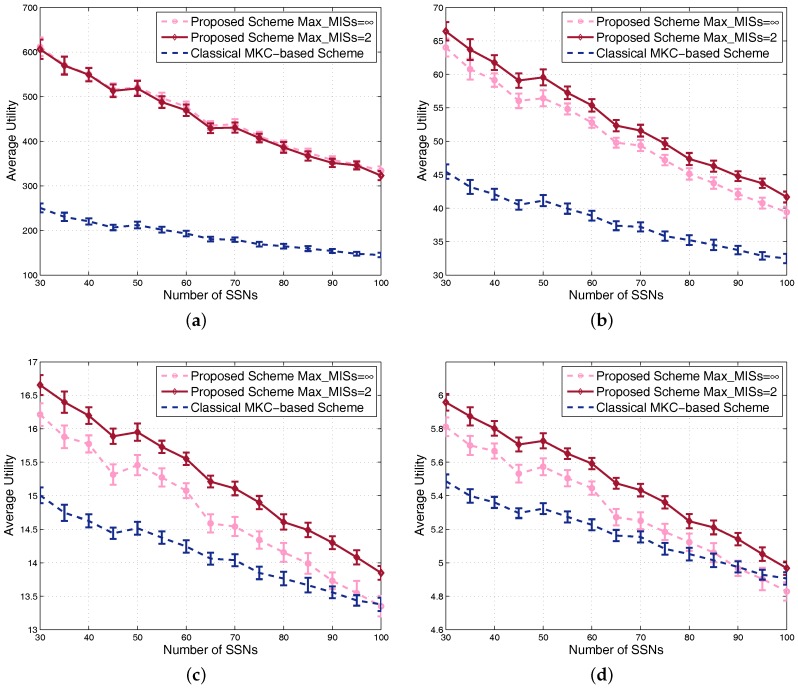
Comparison of utility in dense scenarios: (**a**) α=0; (**b**) α=0.4; (**c**) α=0.8; (**d**) α=1.

Firstly, we compare the utilities of the two schemes along with the increment of the number of SSNs. For different α values, the utilities might be different in the orders of magnitude, so we draw the curves for α=0,0.4,0.8,1 in the four sub-figures in Figure 9 to clearly show the gap between the two schemes. For each sub-figure, we can see that the utilities gradually decrease along with the increment of system density, because the increment of the density brings about more interference. When α is small, our scheme obviously outperforms the MKC-based scheme, especially for dense scenarios. When α is large, our scheme degrades utility to guarantee fairness, so the curves of the two schemes become close. Meanwhile, we also compare the curves with different Max_MISs values and draw the curves with Max_MISs=2 and Max_MISs=∞ in the figures. Max_MISs=2 means searching for two MISs in the central part, so there are at most five groups, i.e., leader set, leader adjacent set, two MISs and the rest. Max_MISs=∞ means dividing the central part into multiple MISs until no vertex is left.

We can see that the performance may not increase by dividing the vertices into more groups. The reason is mainly as follows: more divided MISs correspond to the division of the whole resource into more portions. A group with only a few vertices can only get a small amount of resource leading to very poor performance. By contrast, a group with a large number of vertices can get a large amount of resource, and they are not interfered by their neighbors because their neighbors are not in the same MIS, which leads to much better performance for them than putting them together with their neighbors in the same MIS. In a word, dividing the vertices into more MISs makes the performance difference among vertices increase, hence decreasing the fairness of the system. Since the calculation of system utility integrates fairness consideration, it is not difficult to understand that the achieved system utility may decrease when Max_MISs becomes large. Actually, we also evaluate the performance for Max_MISs equal to different values, such as four and six, but we find that the obtained curves almost overlap with the curves of Max_MISs=2, indicating that the latter corresponds actually to a sufficient number of groups for the case with 30–100 SSNs. An important reason for us to choose Max_MISs=2 is that, the smaller the Max_MISs value, the shorter the simulation time cost required. Based on the above results and analysis, we conclude that, as long as a proper Max_MISs is set, the proposed scheme would be almost better than the classical MKC-based scheme in terms of system average utility.

**Figure 10:**
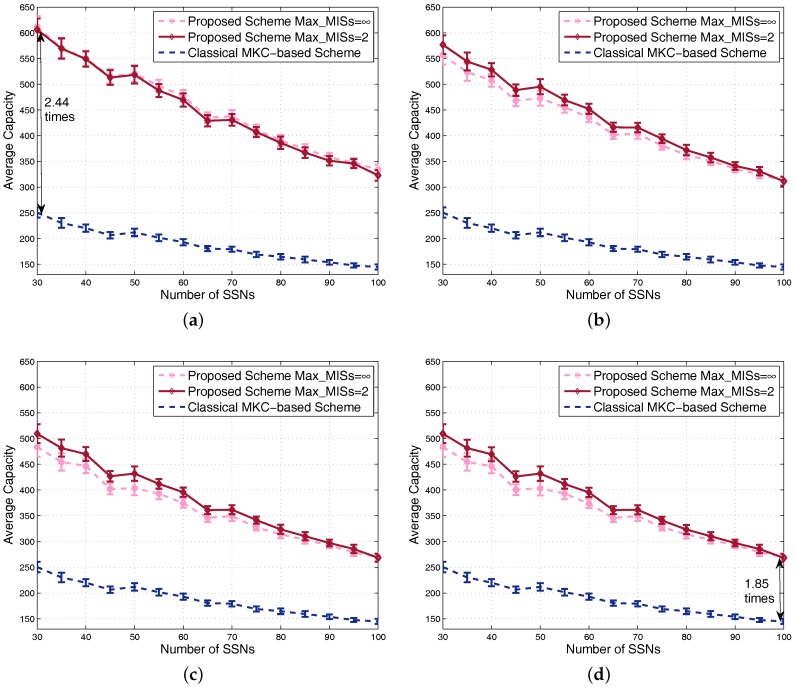
Comparison of capacity in dense scenarios: (**a**) α=0; (**b**) α=0.4; (**c**) α=0.8; (**d**) α=1.

Capacity is evaluated and shown by the four sub-figures of Figure 10. Along with the increment of the number of SSNs, system average capacity decreases due to the increment of interference, which is similar to the feature of utility. Due to the fact that the classical MKC-based scheme is unrelated to the α-fairness factor, its achieved capacity is the same for different α values, so the curves of this scheme in different subfigures are the same. By contrast, the achieved capacity of our proposed scheme gradually decreases with regard to the increment of α because of the increasing tradeoff affect between capacity and fairness. Seen from these subfigures, we conclude that the proposed scheme achieves always better system average capacity than the classical MKC-based scheme, for various α, Max_MISs and numbers of SSNs. For example, the almost worst case of our scheme is at α=1 and the number of SSNs equal to 100, but our scheme still achieves around 1.85-times the system capacity of the classical MKC-based scheme. For the almost best case of our scheme at α=0 and the number of SSNs equal to 30, the achieved capacity of our scheme is around 2.44-times the classical MKC-based scheme. Note that, although the number of scenarios for each point is only half of the previous figures, the confidence intervals in Figure 9 and Figure 10 are not wider than those in previous figures; that is because the scenarios averaged for each point here have the same number of SSNs leading to similar performance results.

**Figure 11:**
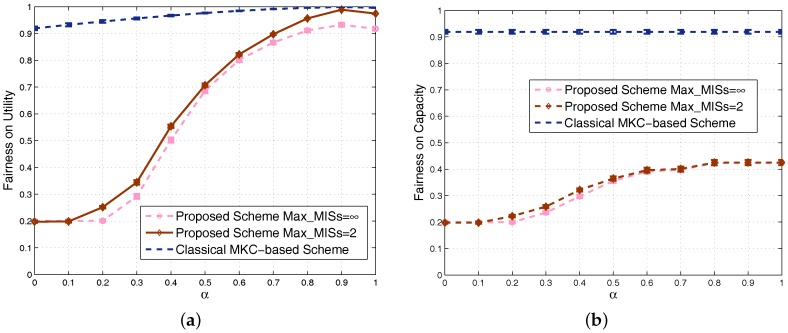
Comparison of fairness in dense scenarios: (**a**) based on utility; (**b**) based on capacity.

We also study the Jain’s fairness index in dense scenarios and obtain Figure 11a,b for fairness based on utility and capacity, respectively. To show the fairness feature in dense scenarios, each point on any curve is obtained by averaging the results of 100 randomly-generated scenarios with 80–100 SSNs. The trends of the curves are similar to the nondense scenarios. Since different sets have the same number of SSNs in the classical MKC-based scheme, its achieved fairness is still high in dense scenarios. Our proposed scheme allocates more resources to the groups with more SSNs, so its achieved fairness becomes obviously low in dense scenarios. By increasing the fairness factor α, our scheme’s fairness on utility could increase to almost the same as the classical MKC-based scheme, but the fairness on capacity cannot be close to it due to the fact that capacity is a metric not integrating α-fairness. One of our future works is to look for schemes to further improve the fairness on capacity. As we know that system capacity and its fairness are two metrics usually opposite of each other, so this future work generally lead to a totally different scheme satisfying fairness and time cost requirements, but sacrificing system capacity, which is out of the scope of this article. Since the 100 scenarios generated between 80 and 100 SSNs for this figure are dense, their achieved performance results are relatively closer than the 100 scenarios in Figure 8. Therefore, the confidence intervals in this figure become much narrower and even almost invisible for many points.

**Figure 12:**
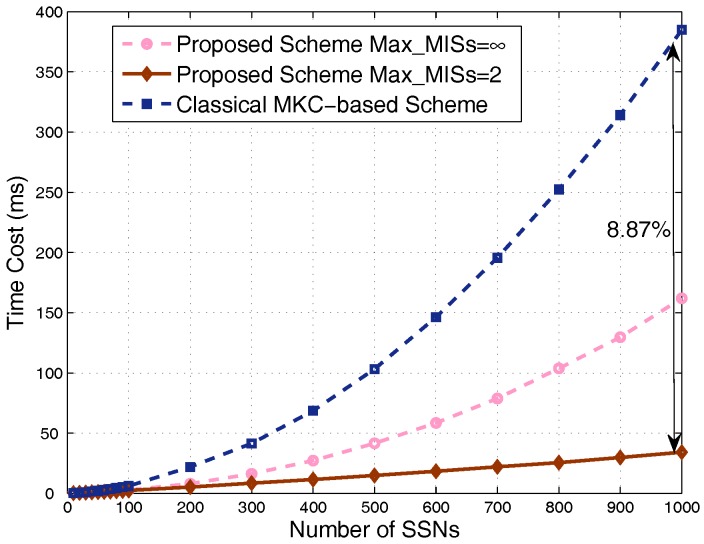
Comparison of time cost in dense scenarios.

Finally, comparison of the time cost is shown in Figure 12. We can see that our scheme is obviously faster than the MKC-based scheme, especially for dense scenarios. By taking Max_MISs=∞, corresponding to the largest simulation time cost of our scheme (the worst case in terms of time cost), the proposed scheme is still obviously faster than the classical MKC-based scheme. We also take Max_MISs=2, the proper value corresponding to the case where the proposed scheme significantly outperforms the classical MKC-based scheme in terms of utility and capacity based on the simulation results above; the time cost dramatically decreases to a very low value, around 8.87% of the time cost of the classical MKC-based scheme in hyper-dense scenarios.

By carefully analyzing the time costs of various parts of the two schemes in the simulation codes, we explicitly see that the key part deciding the complexity of the proposed scheme is the removal operation of nodes in an obtained MIS from the current graph. The complexity of this operation is O(N), leading to a complexity of O(N) for the proposed scheme with Max_MISs=2, which repeats this removal operation only for limited times. For the proposed scheme with Max_MISs=∞, the original graph is divided into many MISs until no node is left. The computational cost is calculated as the sum of arithmetic series when the numbers of nodes in different MISs are identical, although they are generally different. Therefore, its computational complexity can be approximately obtained as $O(N^{2})$. The complexity of the classical MKC-based scheme is mainly decided by the interference calculation and comparison process because the total interference of each node put into any of the *K* sets should be calculated, and this total interference includes interference from all the nodes in other sets. This process makes the complexity of the scheme $O(KN^{2})$, and *K* is related to *N* because we consider the same number of sets for this scheme as the number of MISs for the proposed scheme to guarantee a fair performance comparison. To summarize, in dense scenarios, our scheme achieves a much lower time cost and obviously higher average capacity and utility, without degrading much of the fairness.

## 5. Conclusions

The upcoming application requirements, such as artificial intelligence, smart cities and telemedicine, bring about new networking paradigms for future wireless and mobile communication systems, i.e., the clustering of sensors and the deployment of dense WiFi hotspots and femtocells, uniformly called SSNs in this article. These scenarios require a very fast resource allocation scheme, which guarantees a relatively good system total capacity and utility for transmissions of these applications, while existing schemes, such as the classical MKC-based scheme evaluated in the simulations, do not satisfy this requirement, especially due to their high time cost. A graph-based scheme was proposed in this article, which applied the MIS concept to divide the SSNs into interference-free groups, and resources were allocated to these groups based on a procedure containing the search of several MISs with empirical parameter settings. Simulations demonstrated that the proposed scheme outperformed many other schemes. Because of the low time cost feature of Algorithm 1 for obtaining one random MIS, the proposed scheme achieves the most important advantage (i.e., low time cost), making it quite suitable for usage in future dense networks. Meanwhile, its achieved system capacity and utility are also higher than the classical MKC-based scheme and almost close to the near-optimum obtained by a time-consuming simulated annealing search. By setting a large α-fairness value in the proposed scheme, its achieved fairness of utility can be guaranteed. Our work in the near future is to derive more distance thresholds for various scenarios as the Appendix because suitable distance thresholds could further decrease the time cost. Another future work may be research on new fast schemes with high fairness on capacity to fit both the fairness and time cost requirements.

## Figures and Tables

**Figure A1:**
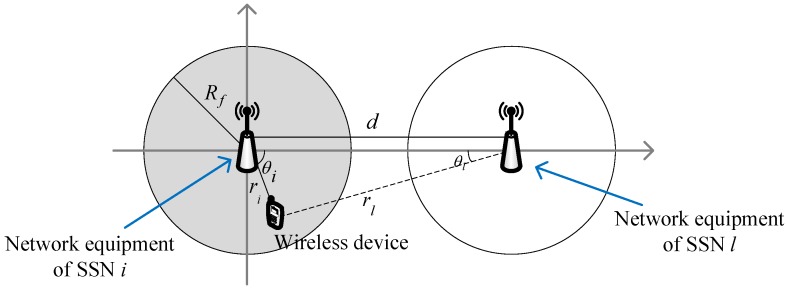
Two-SSN scenario with d>R.

**Figure A2:**
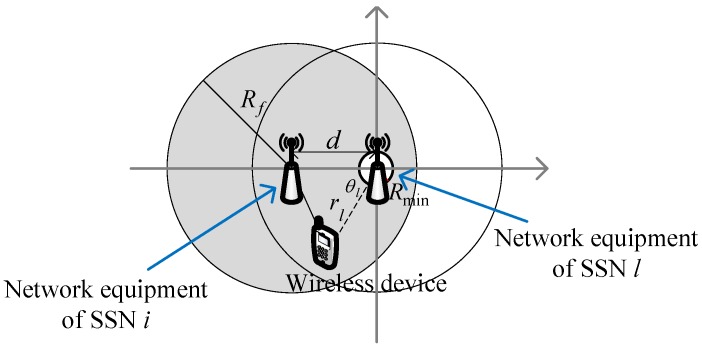
Two-SSN scenario with $d\leq R-R_{min}$.

## Appendix A

This Appendix explains the derivation of the distance threshold $d_{th}$ between two adjacent SSNs. Each of them forms a circular coverage with the network equipment in the center. When the distance is larger than this threshold, they can use the same resource and tolerate the interference. Otherwise, to maximize the total capacity of the two SSNs, it is better to use orthogonal resources for them.

Let us consider the case in Figure A1, where the network equipments of the two SSNs $SSN_{i}$ and $SSN_{l}$ have a distance *d* and the coverage radius of each SSN is *R*. To calculate the downlink interference from $SSN_{l}$ to $SSN_{i}$, the receiving device of the interference could be anywhere in the coverage area of $SSN_{i}$, so we should take the integral of this coverage area. To complete this derivation, we denote the distance as $r_{i}$ between the network equipment of $SSN_{i}$ and a given wireless device accessing it, while the distance between this device and the network equipment is $r_{l}$. The angle between $r_{i}$ and *d* is $\theta_{i}$, while the angle between $r_{l}$ and *d* is $\theta_{l}$.

Based on the relationship between *d* and *R*, the derivation of the interference from $SSN_{l}$ to $SSN_{i}$ can be divided into two cases as follows. In case d>R, as shown by Figure A1, $SSN_{l}$ is not within the coverage of $SSN_{i}$, so the integral for calculating the interference can be written in a normal way. We set the original point of the coordinate system at the network equipment of $SSN_{i}$, so the average interference is calculated by taking the integral within the coverage area of $SSN_{i}$, given by:

(A1) $$\begin{array}{cc} I_{l}= & \frac{P_{t}}{\pi R^{2}}\int_{0}^{R}r_{i}dr_{i}\int_{0}^{2\pi }\frac{1}{r_{i}^{2}+d^{2}-2r_{i}d\cos\theta_{i}}d\theta_{i}\\ = & \frac{P_{t}}{\pi R^{2}}\int_{0}^{R}r_{i}dr_{i}\int_{-\pi }^{\pi }\frac{\frac{1}{r_{i}^{2}}}{1+{\left(\frac{d}{r_{i}}\right)}^{2}-2\frac{d}{r_{i}}\cos\theta_{i}}d\theta_{i} \end{array}$$

Let $t=\tan\frac{\theta_{i}}{2}$; the above integral becomes:

(A2) $$\begin{array}{cc} I_{l}= & \frac{P_{t}}{\pi R^{2}}\int_{0}^{R}r_{i}dr_{i}\frac{1}{r_{i}^{2}}\int_{-1}^{1}\frac{\frac{2}{1+t^{2}}}{1+{\left(\frac{d}{r_{i}}\right)}^{2}-2\frac{d}{r_{i}}\cdot \frac{1-t^{2}}{1+t^{2}}}dt\\ = & \frac{P_{t}}{\pi R^{2}}\int_{0}^{R}r_{i}\frac{2\pi }{\left(d+r_{i}\right)\left(d-r_{i}\right)}dr_{i}\\ = & \frac{P_{t}}{R^{2}}\int_{0}^{R}\left(\frac{1}{d-r_{i}}-\frac{1}{d+r_{i}}\right)dr_{i}\\ = & \frac{P_{t}}{R^{2}}\ln\frac{d^{2}}{d^{2}-R^{2}} \end{array}$$

In the case $d\leq R-R_{min}$, as shown in Figure A2, $SSN_{l}$ is within the coverage of $SSN_{i}$, so the method of integral used in the above case is no longer suitable. One reason is that the interference source $SSN_{l}$ inside the coverage of $SSN_{i}$ making the integral trend be infinite, so we should consider a reference distance $R_{min}$ as the minimum possible distance between the network equipment and any device. Note that, in wireless communication theory, it is quite common to define such a reference distance when calculating the channel propagation. Another reason is that the usage of reference distance $R_{min}$ forms a small circle within the coverage of $SSN_{i}$, making it difficult to take the integral using the previous coordinate system even through the subsection integral may be used. Therefore, for the second case, we build a coordinate system with the original point at the network equipment of $SSN_{l}$ and derive the average interference as follows. Note that, although the case is for $d\leq R-R_{min}$, it is actually equivalent to d≤R, due to the fact that $R_{min}$ should be a very small value compared with *R*, so our discussion in this Appendix covers all the cases.

The edge of $SSN_{i}$ is a circle whose equation under the polar coordinate system can be written as:

(A3) $${\left(s\cos\theta_{l}+d\right)}^{2}+{\left(s\sin\theta_{l}\right)}^{2}=R^{2},$$

which is a quadratic equation with one unknown and can be solved as:

(A4) $$s=\sqrt{R^{2}-d^{2}\sin^{2}\theta_{l}}-d\cos\theta_{l}.$$

Note that the other solution has a negative value, which cannot be used to represent distance. Up to now, we could describe the integral for the average interference as:

(A5) $$\begin{array}{cc} I_{l}= & \frac{1}{\pi R^{2}}\int_{0}^{2\pi }d\theta_{l}\int_{R_{min}}^{\sqrt{R^{2}-d^{2}\sin^{2}\theta_{l}}-d\cos\theta_{l}}P_{t}\cdot \frac{1}{r_{l}^{2}}\cdot r_{l}dr_{l}\\ = & \frac{P_{t}}{\pi R^{2}}\int_{0}^{2\pi }\left[\ln\left(\sqrt{R^{2}-d^{2}\sin^{2}\theta_{l}}-d\cos\theta_{l}\right)-\ln R_{min}\right]d\theta_{l}\\ = & \frac{2P_{t}}{\pi R^{2}}\int_{0}^{\pi }\left[\ln(\sqrt{R^{2}-d^{2}\sin^{2}\theta_{l}}-d\cos\theta_{l})\right]d\theta_{l}-\frac{P_{t}}{R^{2}}\ln R_{min}^{2} \end{array}$$

Let $z_{1}=\int_{0}^{\pi }\left[\ln(\sqrt{R^{2}-d^{2}\sin^{2}\theta_{l}}-d\cos\theta_{l})\right]d\theta_{l}$; we have:

(A6) $$\begin{array}{cc} z_{1}= & \int_{0}^{\pi }\left[\ln(\sqrt{R^{2}-d^{2}\sin^{2}\theta_{l}}-d\cos\theta_{l})\right]d\theta_{l}\\ = & \int_{0}^{\pi }\left[\ln\frac{\left(\sqrt{R^{2}-d^{2}\sin^{2}\theta_{l}}-d\cos\theta_{l}\right)\left(\sqrt{R^{2}-d^{2}\sin^{2}\theta_{l}}+d\cos\theta_{l}\right)}{\sqrt{R^{2}-d^{2}\sin^{2}\theta_{l}}+d\cos\theta_{l}}\right]d\theta_{l}\\ = & \int_{0}^{\pi }\left[\ln\frac{R^{2}-d^{2}}{\sqrt{R^{2}-d^{2}\sin^{2}\theta_{l}}+d\cos\theta_{l}}\right]d\theta_{l}\\ = & \pi \ln\left(R^{2}-d^{2}\right)-\int_{0}^{\pi }\left[\ln(\sqrt{R^{2}-d^{2}\sin^{2}\theta_{l}}+d\cos\theta_{l})\right]d\theta_{l} \end{array}$$

Let $z_{2}=\int_{0}^{\pi }\left[\ln(\sqrt{R^{2}-d^{2}\sin^{2}\theta_{l}}+d\cos\theta_{l})\right]d\theta_{l}$; we take $\theta_{l}=\pi -\phi$ into $z_{2}$ and obtain:

(A7) $$\begin{array}{cc} z_{2}= & \int_{0}^{\pi }\left[\ln(\sqrt{R^{2}-d^{2}\sin^{2}\left(\pi -\phi \right)}+d\cos\left(\pi -\phi \right))\right]d\left(\pi -\phi \right)\\ = & \int_{0}^{\pi }\left[\ln(\sqrt{R^{2}-d^{2}\sin^{2}\phi }-d\cos\phi )\right]d\phi =z_{1} \end{array}$$

Combining (A6) and (A7) above, we get:

(A8) $$z_{1}=\frac{\pi }{2}\ln\left(R^{2}-d^{2}\right).$$

Take (A8) into (A5); we get the average interference as:

(A9) $$I_{l}=\frac{P_{t}}{R^{2}}\ln\left(\frac{R^{2}-d^{2}}{R_{min}^{2}}\right).$$

To derive the thresholds for the two cases, the average receiving power of the useful signal from $SSN_{i}$ to its devices is needed. A reference distance $R_{min}$ around the network equipment of $SSN_{i}$ is also needed; otherwise, this maximal power becomes infinite. Then, we obtain the average receiving power as:

(A10) $$\begin{array}{cc} P_{r}= & \frac{1}{\pi R^{2}}\int_{0}^{2\pi }\int_{R_{min}}^{R}P_{t}r^{-2}\cdot rdrd\theta_{i}\\ = & \frac{P_{t}}{\pi R^{2}}\int_{0}^{2\pi }\ln\left(\frac{R}{R_{min}}\right)d\theta_{i}\\ = & \frac{P_{t}}{\pi R^{2}}\ln\left(\frac{R^{2}}{R_{min}^{2}}\right) \end{array}$$

After obtaining the expressions for the average useful power and the average interference, we can now derive the distance threshold, which is the distance with equal total capacities by orthogonal and non-orthogonal resource allocations, given by:

(A11) $$C_{B/2}=C_{B},$$

where $C_{B/2}$ represents the total capacity of the two SSNs using orthogonal resource allocation and $C_{B}$ represents the usage of non-orthogonal resource allocation. The two total capacities can be written as:

(A12) $$C_{B/2}=C_{i}+C_{l}=B\log_{2}\left(1+SINR_{1}\right),\mathrm{and}$$

(A13) $$C_{B}=C_{i}+C_{l}=2B\log_{2}\left(1+SINR_{2}\right),$$

where $SINR_{1}=P_{r}/\left(I_{l}+N_{0}\right)$, $SINR_{2}=P_{r}/N_{0}$, and *B* represents the total bandwidth of each SSN can use. Taking (A12) and (A13) into (A11), we obtain:

(A14) $$I_{l}=\sqrt{P_{r}N_{0}+N_{0}^{2}}.$$

By taking (A14) into (A2) and (A9), respectively, we obtain the thresholds for the first and the second cases as follows:

(A15) $$d_{th,1}=R\sqrt{\frac{H}{H-1}},\mathrm{and}$$

(A16) $$d_{th,2}=\sqrt{R^{2}-HR_{min}^{2}}.$$

where $H=e^{R^{2}\sqrt{P_{r}N_{0}+N_{0}^{2}}/P_{t}}$. Note that $N_{0}$ is a very small value, so *H* should be slightly larger than one. We have:

(A17) $$\begin{array}{cc} d_{th,2}= & \sqrt{R^{2}-HR_{min}^{2}}\\ > & \sqrt{R^{2}-R_{min}^{2}}=\sqrt{\left(R-R_{min}\right)\left(R+R_{min}\right)}\\ > & \sqrt{\left(R-R_{min}\right)\left(R-R_{min}\right)}=R-R_{min}, \end{array}$$

which is a paradox for the initial condition of this case $d\leq R-R_{min}$. Therefore, (A16) is not a solution to the distance threshold. To sum up, the second case discussed does not reach a solution, so (A15) obtained in the first case is the only solution for the derivation of the distance threshold.
